# Proceedings of the Medical Society of the County of Erie

**Published:** 1886-02

**Authors:** Edward Clark

**Affiliations:** Secretary


### PROCEEDINGS OF THE MEDICAL SOCIETY OF THE COUNTY OF ERIE.

*Annual Meeting, January 12, 1886.*

The annual meeting of the Medical Society of the County of Erie was held January 12, 1886, at the Y. M. C. A. building. The meeting was called to order at 10.30 a. m., by the

President, Dr. J. B. Andrews, and the following members were in attendance: Drs. Nott, Howell, Haberstro, Hubbell, Crego, Thornton, Persons, Porter, Walsh, Fowler, Brown, Johnson, Harvey, Gumaer, Geo. Abbott, F. W. Abbott, Park, Howe, J. C. Greene, W. D. Greene, S. S. Greene, Dorland, Jackson, Bidaman, Barker, Samo, Howard, Lynde, Tobie, Putnam, Wilson, Wm. Ring, C. A. Ring, Wm. G. Ring, Barnes, Frederick, W. W. Potter, J. H. Potter, Banta, Lothrop, B. G. Long, E. H. Long, E. Storck, Bartow, Mickle, Dagenais, Briggs, Hayd, O'Brien, Daggett, Campbell, Hopkins, Earl, Boies, O. C. Strong, Kamerling, Phelps, Mann, Wyckoff, Coakley, B. P. Hoyer, Vandenburgh, Gail, Hill, Brecht, Dambach, Wall, Moody, Folwell, Boardman, Cronyn, Hartwig, Meisburger and Clark, and, by invitation, Drs. Johnstone, Whitnall, Bode, Grosvenor, Callanan, Dewitt C. Greene, Starr, Gager, Rich, Morrison.

The minutes of semi-annual meeting, and those of special meeting, were read and approved.

The Committee on Membership reported as follows:

BUFFALO, January 12, 1886.

*Mr. President and Members of the Medical Society of the County of Erie:*

Your Committee on Membership respectfully report that, at the last regular meeting of the Society, the following-named gentlemen made the prescribed application for membership in this Society: Dr. J. W. Grosvenor, graduate of New York University Medical College; Dr. H. W. Bode, graduate of Würzburg, Germany; Dr. F. M. Rich, graduate of Bellevue Hospital Medical College; Dr. D. A. Morrison, graduate of Medical Department, University, Iowa; Dr. De Lancey Rochester, graduate of Medical Department, University, Buffalo; Dr. J. M. Stanley, graduate of Medical Department, University, City of New York; Dr. H. G. Whitnall, graduate of Medical Department, University, City of New York; Dr. E. E. Johnstone, graduate of Medical Department, University, Buffalo; Dr. Dewitt C. Greene, graduate of Medical Department, University, Buffalo.

Having examined the credentials and status of the applicants, and finding them entirely satisfactory, your committee recommend that they be elected to membership.

THOS. M. JOHNSON,
*Chairman of Membership Committee.*

On motion of Dr. W. W. Potter the report was adopted, and the new members introduced to the Society.

Applications for membership were received from the following gentlemen, which were referred to the Committee on Membership, viz.: Wm. C. Callanan, Thos. M. Crowe, Arthur W. Hurd, Edwin H. Norton, Edward T. Smith, Mark N. Brooks, Wm. A. McFarlane, Henry H. Bingham, Benj. W. Cornwell, Thos. F. Dwyer, Elmer Starr, Edward L. Gager, John T. Pitkin.

At this point, Dr. Andrews, the retiring President of the Society, read his valedictory address, taking for his subject "The Increase of Insanity." The paper was an admirable one, and was listened to throughout with attention. At its close, on motion of Dr. W. W. Potter, the thanks of the Society were tendered Dr. Andrews for his interesting and instructive address.

Under the head of "Reports of Committees and Officers," Dr. E. T. Dorland, who was appointed to prepare a suitable memorial of the late Dr. Geo. H. Lapham, reported, and it was unanimously adopted.

On motion of Dr. Wm. Ring, the Secretary was authorized to send a copy of Dr. Lapham's memorial to the State Society for publication in its transactions.

Dr. W. H. Gail, a committee appointed to prepare a suitable memorial of Dr. Jabez Allen, presented the same, which was unanimously adopted, and, on motion of Dr. W. W. Potter, was ordered placed on file, and a copy sent to the family of the deceased, and furnished to the medical press for publication.

Dr. Fowler presented a memorial of the late Dr. C. A. McBeth, which was ordered spread on the minutes.

Dr. E. Storck, Chairman of the Board of Censors, reported that since the semi-annual meeting the Board had not found it necessary to prosecute any illegal practitioners, but that two, viz., "Drs. Retel and Dirsch," were practicing without the proper qualifications, and had been notified to cease practicing until they had complied with the requirements of the law. Mrs. Matteson, the clairvoyant, had been taken into the police court, and also before the grand jury, but for want of sufficient evidence she was not indicted.

Dr. Storck said if any member of the Society could procure positive evidence against her, that the Board would try to prosecute her, as she is absolutely without any authority to practice.

The Board wished also to call the attention of the Society to the fact that under existing laws every physician must become a member of a county society, in order to fully comply with the legal requirements.

On motion of Dr. Coakley, the Board were given power, if necessary, to employ counsel to prosecute illegal physicians, and they were also instructed to investigate the laws relating to the necessity of physicians becoming members of county medical societies, and to report at next meeting.

Committee on Legislation reported as follows: That they had been unable to get legislation on the bill recommended by this Society last year. This bill, as the Society would remember, took away from the teaching bodies all power to grant a license to physicians to practice. They would, therefore, suggest that the bill which was prepared by the State Society last year be sent to our Senator and Members of the Assembly, with the request that they introduce the same, and use all honorable means to pass it. This bill is almost identical with our former bill, with the exception that it allows three members out of a total nine of the Board to hold positions in medical colleges.

20

On motion of Dr. Wm. Ring, the report of Committee on Legislation was received.

Dr. J. C. Greene moved that the recommendations contained in the report be adopted, which was carried.

Dr. J. B. Samo, the Librarian of the Society, presented his report, which, on motion, was received and placed on file.

He reported that the library had been returned to the Buffalo Medical College, and requested that he be empowered to purchase missing volumes of the State Society's Reports.

In regard to the Librarian's suggestion relative to purchasing volumes of State Societies transactions to fill up broken sets now in library, the subject matter was referred to a committee, consisting of Drs. Samo and Mann, said committee to have power to exchange certain volumes, and to purchase those necessary to complete broken sets.

The Treasurer of the Society, Dr. F. W. Abbott, presented his annual report, which, on motion of Dr. Haberstro, was referred to the Auditing Committee. The Chair named as such committee Drs. O'Brien, Boardman and J. Cronyn. The Committee reported that they found the accounts correct.

Dr. Andrews said he would re-appoint the present Legislative Committee, as they seemed to take an interest in the work. It consists of Drs. J. C. Greene, E. Storck, M. D. Mann, F. S. Crego, A. R. Davidson, A. H. Briggs, H. R. Hopkins.

Dr. Howe, from Committee on Essayists, reported that they had invited Dr. F. W. Hinkel to give the Society an address.

On motion, the report was received and adopted.

Dr. Barker, from Committee on Revision of By-Laws, presented a written report of intended amendments to the by-laws, and moved that the Secretary be instructed to have them printed in pamphlet form, a copy to be sent to each member of the Society for his consideration.

On motion, the report was received and recommendations contained therein adopted.

Under the head of "Election of Officers," Dr. J. C. Greene moved that the Chair appoint a Nominating Committee to nominate officers to be elected by this Society.

Dr. Hopkins offered as an amendment, that the committee nominate both officers and delegates. The motion, as amended, was carried.

The Chair appointed as a Nominating Committee, Drs. J. C. Greene, Briggs and Howe.

While the Nominating Committee were making up a list of officers, the Society opened under the head of "Miscellaneous Business." Under this head Dr. Park offered the following resolution, which was unanimously adopted:

WHEREAS, Messrs. Foote & Swift, of Philadelphia, have memorialized the Secretary of State of the United States to obtain, through diplomatic correspondence, a digest of the laws of all foreign nations bearing on the matter of State control of medical education and of the practice of medicine, and have also requested the Attorneys-General of the different States at home to the same effect concerning the laws of each State, with a view to publishing the information thus obtained in concise form.

*Resolved*, That the Erie County Medical Society of New York heartily endorse such an endeavor to publish information so desirable in accessible form, and consider the effort a commendable one.

Dr. Crego moved that the Board of Censors be instructed to determine whether a member of this Society can join a homeopathic medical society and still retain his membership in this Society, and whether he is liable to expulsion for such action. Carried.

Dr. E. Storck moved that the Board of Censors be empowered to determine whether a person who dealt in patent medicines, nostrums, etc., can still hold his membership in this Society. Carried.

A communication from the State Medical Society, relating to the purchasing of its transactions, was read, and ordered placed on file.

Dr. W. W. Potter moved that an appropriation be made to purchase twenty-five volumes of the transactions of the State [illegible]ciety, which was lost.

At this point, the Nominating Committee reported that they had made the following nominations:

For President, Dr. E. T. Dorland; Vice-President, Dr. O. C. Strong, of Colden; Secretary, Dr. Edward Clark; Treasurer, Dr. F. W. Abbott; Librarian, Dr. J. B. Samo; Committee on Membership, Drs. T. M. Johnson, Lucien Howe, J. W. Putnam; Censors, Drs. E. Storck, Hopkins, Briggs, Van Peyma and Gail; Delegates to State Medical Society, Drs. Hubbell, Mann, Bourne, Hinkel, Howell.

Dr. Hopkins moved that the report of the Nominating Committee be received. Carried.

Dr. Hopkins moved that the Secretary cast the ballot of the Society for Dr. Dorland for President, which was carried, and Dr. Dorland was declared unanimously elected President for the ensuing year.

On motion, the Secretary cast the ballot of the Society for Dr. O. C. Strong for Vice-President, and Dr. Strong was unanimously elected Vice-President for the ensuing year.

Dr. Clark declined renomination for Secretary, and, on motion, he was directed to cast the ballot of the Society for Dr. Wm. H. Thornton for Secretary. Dr. Thornton was declared unanimously elected Secretary for the ensuing year.

Dr. F. W. Abbott was unanimously re-elected Treasurer, and Dr. J. B. Samo was unanimously re-elected Librarian, the Secretary casting the ballot of the Society for both.

The Secretary was directed to cast the ballot of the Society for Drs. Johnson, Howe and Putnam for a Committee on Membership, and they were declared unanimously elected for the ensuing year.

The Secretary was directed to cast the ballot of the Society for Drs. Storck, Hopkins, Briggs, Van Peyma and Gail, for Board of Censors, and they were declared unanimously elected for the ensuing year.

Dr. Hopkins moved to proceed to ballot for delegates to the State Medical Society, which was carried, the Chair appointing as tellers Drs. E. H. Long and F. R. Campbell.

On motion, the delegates were voted for singly. Eight ballots were taken, resulting in the election of the following-named gentlemen as delegates to the State Medical Society for the four years ending January 1, 1890, viz: Drs. B. Bartow, F. S. Crego, M. B. Folwell, F. W. Hinkel, and C. W. Bourne of Hamburg.

On motion of Dr. Bartlett, the thanks of the Society were tendered to Dr. Andrews, the retiring president. Dr. E. T. Dorland, the President-elect was duly inducted into office. He thanked the members for the great honor conferred upon him in calling him to a position which had been held by so many eminent men. He asked the co-operation and indulgence of the members in the discharge of his duties, after which the Society adjourned.

EDWARD CLARK, *Secretary.*

